# Practical lessons for bringing policy-makers on board in sexual and reproductive health research

**DOI:** 10.1186/s12913-016-1889-1

**Published:** 2016-11-11

**Authors:** Aurore Guieu, Wei-Hong Zhang, Yves Lafort, Peter Decat, Sara De Meyer, Shuchen Wang, Birgit Kerstens, Els Duysburgh

**Affiliations:** 1 International Centre for Reproductive Health, Ghent University, De Pintelaan 185, UZP114, Ghent, 9000 Belgium; 2 MOMI consortium, http://www.momiproject.eu; 3 INPAC consortium, http://www.inpacproject.eu; 4 CERCA consortium, http://www.icrh.org/project/cerca-community-embedded-reproductive-health-care-adolescents-latin-america; 5 DIFFER consortium, http://www.differproject.eu

**Keywords:** Stakeholders, Policy-makers, Research Translation, Policy, Sexual and Reproductive Health, Latin America, Sub-Saharan Africa, China, India

## Abstract

**Background:**

The need to translate research into policy, i.e. making research findings a driving force in agenda-setting and policy change, is increasingly acknowledged. However, little is known about translation mechanisms in the field of sexual and reproductive health (SRH) outside North American or European contexts. This paper seeks to give an overview of the existing knowledge on this topic as well as to document practical challenges and remedies from the perspectives of researchers involved in four SRH research consortium projects in Latin America, sub-Saharan Africa, China and India.

**Methods:**

A literature review and relevant project documents were used to develop an interview guide through which researchers could reflect on their experiences in engaging with policy-makers, and particularly on the obstacles met and the strategies deployed by the four project consortia to circumvent them.

**Results:**

Our findings confirm current recommendations on an early and steady involvement of policy-makers, however they also suggest that local barriers between researchers and policy-making spheres and individuals can represent major hindrances to the realization of translation objectives. Although many of the challenges might be common to different contexts, creating locally-adapted responses is deemed key to overcome them. Researchers’ experiences also indicate that - although inevitable - recognizing and addressing these challenges is a difficult, time- and energy-consuming process for all partners involved. Despite a lack of existing knowledge on translation efforts in SRH research outside North American or European contexts, and more particularly in low and middle-income countries, it is clear that existing pressure on health and policy systems in these settings further complicates them.

**Conclusions:**

This article brings together literature findings and researchers’ own experiences in translating research results into policy and highlights the major challenges research conducted on sexual and reproductive health outside North American or European contexts can meet. Future SRH projects should be particularly attentive to these potential obstacles in order to tailor appropriate and consistent strategies within their existing resources.

**Electronic supplementary material:**

The online version of this article (doi:10.1186/s12913-016-1889-1) contains supplementary material, which is available to authorized users.

## Background

Health system research findings should not be limited solely to academic circles, as existing evidence on the root causes of health system weaknesses and on feasibility, cost-effectiveness, and sustainability of potential interventions can inform health policies, care delivery and health systems. This means that researchers have to find suitable ways of passing their findings on to the key actors who are responsible for shaping agendas and driving policy change while collecting the feedback of those actors. The need to ensure the translation of research results into policy, notably through policy-makers’ involvement, is increasingly apparent in the literature – however, few resources tackle this issue in the context of sexual and reproductive health (SRH) outside North American and European contexts, especially from the perspective of researchers engaged in translating their findings into policy.

The International Centre for Reproductive Health (ICRH) is a research centre embedded into the University of Ghent, Belgium, and coordinates four European Union-funded projects addressing a variety of sexual and reproductive health issues in Latin America, sub-Saharan Africa, China and India in the frame of the European Commission (EC) Seventh Framework programme (FP7). The project duration varies from 4 to 5 years:

- CERCA (Community-Embedded Reproductive Health Care for Adolescents in Latin America)
- DIFFER (Diagonal Interventions to Fast Forward Enhanced Reproductive Health)
- INPAC (Integrating Post-Abortion Family Planning Services into Existing Abortion Services in Hospital Settings in China)
- MOMI (Missed Opportunities in Maternal and Infant Health).

The projects, their objectives, consortium partners, study countries/sites and time frames are described in Table 1.

**Table 1:** Characteristics of four EC-FP7 projects coordinated by ICRH

Project and Timeline	General Objective	Study Sites	Consortium partners
CERCA 1/03/10 – 29/02/14	To contribute to global knowledge about how health systems can be more responsive to the sexual and reproductive health needs of adolescents and, by extension, to other health needs of Latin American populations.	City of Cuenca (Ecuador) City of Managua (Nicaragua) City of Cochabamba (Bolivia)	South Group (Bolivia), University of Cuenca (Ecuador), Lithuanian University of Health Sciences (Lithuania), Amsterdam University (The Netherlands), Centro de Investigaciones y Estudios de Salud (Nicaragua), Instituto Centro Americano de Salud (Nicaragua)
DIFFER 1/10/11 – 30/09/16	To improve sexual and reproductive health for all women by expanding and strengthening sexual and reproductive health services, and providing and testing targeted interventions for female sex workers in the context of existing health systems.	Mombasa County, Coastal Province (Kenya) Cities of Tete and Moatize, Tete Province (Mozambique) City of Durban (eThekwini District), KwaZulu-Natal Province (South Africa) City of Mysore, Karnataka State (India)	Ashodaya Samithi (India), International Centre for Reproductive Health Association (Kenya), International Centre for Reproductive Health Association (Mozambique), University of the Witwatersrand – MatCH (South Africa), University College London – Institute for Global Health (United Kingdom)
INPAC 1/08/12 – 31/07/16	To evaluate the effectiveness of introducing integrated post-abortion family planning services into existing hospital-based abortion services in China in order to reduce unintended pregnancies and repeated abortions.	30 of 31 provinces, municipalities and autonomous regions of mainland China	Chinese Society of Family Planning – Chinese Medical Association (China), Fudan University (China), National Research Institute for Family Planning (China), West China Second University Hospital of Sichuan University (China), University of Aarhus – Danish Epidemiology Science Centre (Denmark), Liverpool School of Tropical Medicine (United Kingdom)
MOMI 1/02/11 – 31/01/16	To improve maternal and newborn health through a focus on the postpartum period, adopting context-specific strategies to strengthen health care delivery and services at both facility and community level in four sub-Saharan countries.	Kaya District (Burkina Faso) Kwale District (Kenya) Ntchisi District (Malawi) Chiuta District (Mozambique)	Institut de Recherche en Sciences de la Santé (Burkina Faso), International Centre for Reproductive Health Association (Kenya), Faculdade de Medicina – Eduardo Mondlane University (Mozambique), International Centre for Reproductive Health Association (Mozambique), Parent and Child Health Initiative (Malawi), Faculdade de Medicina da Universidade do Porto – Department of Hygiene and Epidemiology (Portugal), University College London – Institute for Global Health (United Kingdom)

All four projects aim not only at producing research evidence but also at ensuring that the findings can be translated into policy changes. This aim is illustrated by the fact that in the design phase of each of these projects, specific activities have been foreseen and allocated to enhance this translation process. All FP7 projects group their activities into thematic so-called “work packages”, and the four ICRH-coordinated projects each dedicate a full work package to translation activities, which deliberately include a strong involvement of stakeholders, among others policy-makers. In addition, activities embedded in other work packages may also be considered as related to the translation of research findings into policy.

Stakeholders may be defined as all individuals or organizations having a potential role in influencing policy change. They include notably government actors (at national, provincial, district/county, municipal levels) but also health authorities, donor agencies, non-governmental organisations, professional bodies, and/or communities [1–3]. We focus here on government actors and health authorities, subsequently called ‘policy-makers’, as they retain the main role in decision-making and the four projects have developed specific processes to engage this group.

This paper seeks (1) to give an overview of the currently available knowledge on policy-makers’ involvement in SRH research outside North American and European contexts and on the impact in strengthening health systems and policies, and (2) to document - in the light of the existing knowledge - challenges and remedies encountered in four projects in involving policy-makers to ensure translation of SRH research into action.

## Methods

To answer both our objectives we used a literature review, a project document review and key-informant interviews. In line with objective 1, we aimed through the literature review at highlighting general priority areas in translation activities, and possibly identify practical lessons learnt from other research projects in strengthening health systems and policies rather than providing an extensive and detailed state-of-the-art overview of that literature. Therefore, we chose not to conduct a systematic review. This report, and notably the methodology and results of its interviews below, were written using the COREQ consolidated criteria for reporting qualitative studies [4].

We searched the literature using Web of Science and using the following four selection criteria: (1) literature addressing policy-makers’ involvement (2) in the field of SRH, (3) in contexts outside North American and European settings, particularly in low- and middle-income countries (LMICs), and (4) published after 2006. Simultaneously, information was collected from the four projects on their specific translation strategies, in order to give the necessary background to the realization of objective 2. Information was principally found in project documents describing protocols, objectives and activities, policy and advocacy reports produced by the projects, as well as periodic reports to the funder (EC). This process provided information on the extent to which the involvement of policy-makers was foreseen at the beginning of each project, with the objectives set by each project consortium regarding translation activities.

The results of the literature review, as well as the information collected from the projects, informed the development of an interview guide and were used in order to conduct a set of key informant semi-structured interviews. This interview guide included 13 questions divided in three categories representing project phases: project design, implementation, and lessons learnt (see Additional file 1).

Questions were oriented at researchers’ personal experiences and aimed at highlighting the context in which translation activities were embedded and conducted in the project rather than said activities as such – these were provided in project documents. We sought notably to identify obstacles and barriers in various phases of the projects, and what consequently influences the choice of response strategies to address these. Due to the clear identification of interviewees – the six current ICRH project coordinators of the four projects described above, exception made of the first author - criteria such as sampling, method of approach, non-participation, setting and data saturation are not relevant for this particular study. The first author and interviewer (MSc, female) was part of the project coordinating team for two of the four projects (DIFFER and MOMI) and knew all interviewees prior to the interviews. The study had been previously initiated but paused and no interviews had yet taken place.

Interviews were conducted by the first author, face-to-face when possible; if not, the guides were completed in written form. All interviews were conducted on a one-to-one basis. When answers were not clear in the written documents or more information was desirable, a face-to-face or telephone follow-up discussion was organized. Transcripts were not returned as interviewees were consistently involved in the writing process of this report as co-authors. Responses were then coded by the first author using her interview notes and the Nvivo 10.0 software to identify common and minor themes – themes were not defined beforehand. They were then compared with the results of the literature review in order to confront the project teams’ experiences with the existing knowledge on translation processes. As all data analysed is presented in this report and results organized along the themes which emerged during coding, we have not included the coding tree as such. This research did not require ethical approval within Ghent University.

## Results

### Available scientific knowledge on translating health research into action

We selected 18 academic articles answering a number of our selection criteria described above. The objective of the selection was to uncover existing literature’s main themes to relate them with the experience of project coordinating teams in four ICRH-coordinated projects. Therefore our search was not systematic, a choice reinforced by the following elements. Literature on translating research into policy is extensive, however few references are discussing it in the specific context we were looking at (SRH outside of North American and European contexts, particularly in LMICs). When literature looks at this context, it tends to look at stakeholder involvement with a focus on community and religious leaders and societal change rather than policy-makers and policy change. Only three references answered all four selection criteria [5–7]. Therefore, we had to consider the extent to which other references were approaching the context we wanted to examine, or were discussing translation processes without linking them specifically to other contexts, even when they did not answer all four selection criteria. We were careful to select studies which could inform translatable dynamics to our context. References included were used to answer objective 1 and give an overview of the currently available knowledge on policy-makers’ involvement in our specific context.

Our literature review results show that knowledge management is now recognized as a key component of health system research; it can be defined as the creation and management of linkages between research and action [8]. It was particularly put under the spotlight at the 2004 World Health Organization Ministerial Summit on Health Research in Mexico, which sought to address the so-called “know-do” gap by exploring ways of translating research into action. The final statement of the meeting stresses the need to base *“health policy, public health, and service delivery (…) on reliable evidence derived from high quality research [because] research evidence comes from various sources (…) and measures the benefits and potential risks of health interventions”* [9]. Others call on moral and ethical considerations to support such statements [3].

In the context of knowledge management, ‘action’ is primarily understood as policy change resulting ideally in improved service delivery and ultimately in improved well-being for the populations. Policy change is thus a major objective of research translation efforts. As stated in the introduction, a number of individuals and organizations may be involved in policy change processes. Therefore, depending on the research context, such efforts should target a wide range of these actors, including policy-makers [2, 3, 5]. Because policy change is an increasingly complex and chaotic process [5], influenced by multiple factors (social, economic, political or cultural) [10, 11], researchers should be aware of the competing priorities of policy-makers in their decision-making processes [6]. This has implications in deciding which policy-makers to target, depending on the kind of policy change aimed at: changes in agenda-setting, narratives, institutions, policy or implementation [6, 12]. Ultimately, the objective of this process is to foster advocacy for SRH *“supportive policies”* [13].

Research results being taken up by policy-makers is not an automatic process. It requires a sustained investment since producing scientific evidence, however strong, is often not enough to ensure it is considered in future policy-making [7]. Therefore, strategies have to be proposed in order to *“establish a conducive environment in terms of organizing regular meetings and consultations with key policy bodies”* [6]. Those strategies might consist of push and/or pull efforts to create an *“appetite”* [14] for research in policy-makers and to encourage them to take up evidence when changing policy [14, 15].

To create such enabling fora, significant work should be carried out in order to identify the relevant institutions, networks or individuals [6] and to be prepared to address potential conflicts of interest [2]. Communication is essential to maximize the uptake of research results and framing is its cornerstone, as presenting the results in a clear and concise way increases their responsiveness to policy-makers’ needs [5]. An understanding of those needs and of policy-making processes is critical in order to ensure the best translation of research results. Because of its complexity, the process is particularly challenging. It means that all actors involved need to make efforts in terms of access to data, results framing and dissemination, network strengthening, which means investments in financial, time and human resources [7, 16, 17].

The research community is paying increasingly attention to these processes; however the literature rarely touches upon their specifics in the field of SRH. Because SRH interrogates a number of social norms and goes far beyond a simple public health paradigm, the uptake of even the most technical SRH research can meet significant obstacles [6]. Acknowledging sexual and reproductive health rights remains sensitive and researchers may face specific resistance from policy-makers on topics such as abortion, gender identity and sexual orientation, or sex work [5].

Little also exists on translation processes outside North American and European contexts and more particularly in LMICs [12, 14]. However, researchers wanting to bring their evidence into policy-making do face additional challenges in LMICs, notably because of the *“limited critical mass and absorptive capacity (…) to undertake multiple and competing initiatives”* [8] as well as rapidly evolving social environments [16]. In addition, LMICs offer fewer opportunities to engage with policy-makers in dedicated fora and fewer resources are attributed to SRH [5, 6]. When SRH is targeted in those settings, the literature rather focuses on other stakeholders, especially community and/or religious leaders. Thus, how to engage policy-makers outside North American and European contexts in the field of SRH is rarely touched upon.

Besides those gaps, criticism has been raised about the current paradigm in which translation of research outputs is considered. Academics have warned that although framing results in an understandable way is important, the sole needs of policy-makers should not dictate health system research as they are influenced by public opinion and electoral opportunities [18]. Funding agencies have sometimes been criticized for expecting quick and clear returns on investments in translation activities, a demand that might jeopardize future funding opportunities for SRH research [3, 6].

### Translation strategies used in four FP7 projects

The review of project documents of the four ICRH-coordinated projects showed that the four research consortia put into place a range of strategies to enhance policy-makers’ involvement at different stages of the projects.

A first step all the projects took was to identify existing policies and potential gaps at national and study site levels through a policy analysis. Although the focus at this initial stage was more on policy content and thus not strictly speaking a translation activity, the policy analysis allowed to identify areas where policy change is needed as well as to map which policy-makers should be approached. The characteristics of policy-makers approached for each project are described in Table 2. These policy-makers were interviewed as key informants (DIFFER, INPAC) or consulted in the frame of causal analysis workshops (MOMI), which constituted a first contact with the projects. All projects invited policy-makers to kick-off meetings (DIFFER, MOMI, INPAC), annual consortia meetings (INPAC), or a first individual meeting (CERCA).

**Table 2:** Policy-makers approached

Project	Positions/Institutions	Topics
CERCA	Ministries of Health officials (national, departmental and municipal level) Ministries of Education officials National Health Boards Staff health centres Community level	General SRH Adolescent SRH
DIFFER	Dedicated divisions of Ministries of Health Provincial and District administration (both general and SRH-specific) Existing expert working groups National AIDS Councils	General SRH HIV/AIDS Vulnerable populations
INPAC	National, provincial and district government officials Scientific experts Health managers at different levels and types of hospitals	General SRH Family planning & post abortion contraception
MOMI	District Health Management Teams Provincial Directors for specific divisions Regional Health Directors National Health Institutes Regional Hospital Managers	General SRH Maternal and Newborn Health (MNH)

Throughout the projects, policy-makers have been provided with fora to give feedback on the project objectives and/or on the design, selection and implementation of the interventions at study sites. DIFFER, INPAC and MOMI formed Policy Advisory Boards (PABs) at each study site, meeting at least yearly (DIFFER, MOMI) or bi-yearly (INPAC). The main objective of PABs is to maximize involvement, which is key for the sustainability of project outcomes. PABs also provide ethical and methodology guidance. CERCA chose to convene individual or small group meetings with policy-makers rather than forming boards. In addition to their PABs, DIFFER and MOMI organized stakeholders’ workshops, specifically to collect policy-makers’ (among other stakeholders) feedback on the results of their initial situation analysis and the implications for the interventions.

The four projects also implemented mechanisms to reflect on the translation process and share the research results with policy-makers at the end of the project. As such, the CERCA consortium organized a conference primarily attended by policy-makers, with the objective of disseminating results on teenagers’ SRH. Additionally, three national reference documents were developed by CERCA in which the most important project results and recommendations are mentioned. These were distributed amongst policy makers of the three involved countries (Bolivia, Ecuador and Nicaragua). An evaluation workshop is to be conducted in the frame of DIFFER at the end of the project to discuss opportunities to translate the project outcomes into policy and guidelines to improve female sex workers’ SRH. INPAC aims at developing three specific tools to inform the translational process in the context of SRH (particularly abortion and family planning) in China: a list of potential barriers to translation, recommendations on communicating with PABs, and a strategy for translating INPAC results into actual policy. MOMI will similarly produce policy recommendations in a format adapted to policy-makers’ needs.

### Challenges and solutions

Key informant interviews provided additional information on challenges, solutions and lessons learned regarding policy-makers involvement in project design and implementation.

Key informant interview results show that there is a consensus that involving policy-makers is critical to improve both local ownership and scientific knowledge and to ensure ultimately that health outcomes improve for the target populations. They also show that participating policy-makers were selected based on their familiarity with the project topics and their responsiveness to the first contact.

All interviewees agreed that policy-makers have to be involved early in the project. This is considered the best (although not automatic) guarantee that the involvement remains steady throughout the project, that needs and opportunities are correctly identified, and that the implementation of the interventions would not be met with resistance. Providing information to policy-makers is not considered enough and all projects searched to create effective ways of collecting inputs. This is illustrated by the different strategies described above (workshops, advisory boards, etc.…).

Involving policy-makers proved strenuous. Table 3 shows the main obstacles met and the corresponding solutions experimented in the four projects.

**Table 3:** Main translational challenges and solutions implemented

	Challenges	CERCA	DIFFER	INPAC	MOMI	Solutions
Challenges depending on individual characteristics of policy-makers						Strategies address different challenges simultaneously. Here are listed the most common solutions implemented by the projects to overcome obstacles: 1. Adapt modes of communication, rhythm and language to policy-makers’ needs. 2. Ensure regularity of communication at all stages of the research process. 3. Be coherent with national and local policies. 4. Build on long-term relationships and networks. 5. Encourage the participation of different types of policy-makers. 6. Consider incentives (financial or training) for policy-makers to engage.
1	Lack of motivation to engage in the project	x	x	x	x	
2	Reluctance to share data with project researchers and staff	x		x		
3	Lack of knowledge about research processes and credibility of research results	x	x			
Structural challenges – policy-making structures and local consortia partners						
1	Geographical distance	x	x			
2	Time constraints to engage in the project	x		x	x	
3	Personnel turnover at local and national levels	x		x	x	
4	Lack of weight of the local consortium partner as an organization towards policy-makers	x	x			
5	Translation activities time-consuming and stretching staff capacity for local consortium partners		x			
6	Translation activities frustrating and non-rewarding for local consortium partners		x		x	

The projects all experienced similar challenges in policy-makers’ attitudes and the consortia worked on improving motivation and interest of policy-makers whether in the project itself (CERCA) or in research findings in general (DIFFER). Two projects faced additional obstacles of policy-makers worrying the project would go against national regulations on abortion (INPAC) or being reluctant to participate in a project aiming at sex workers (DIFFER). MOMI was received more positively by policy-makers who recognized the need for enhancing post-partum care.

Geographical distance and high levels of policy-makers’ turnover were the two main structural challenges cited by project coordinators. This required from local consortium partners to regularly repeat efforts to involve policy-makers at a distance or to seek new appointees, which is reflected in local consortium partners feeling that translational activities are time-consuming and rarely rewarding. The strategies to make and keep contact with policy-makers were dependent on the existing networks at the different sites: if in some countries CERCA had to rely on individual mailing because of the lack of such networks, INPAC worked extensively with them, using the presence of local consortium partners in the national and/or local decision-making processes.

Experiences with policy-makers could also be positive, particularly when they agreed with the project objectives, recognized the needs at stake, and were thus eager to participate. This was not only beneficial for the projects but also rewarding for the teams (DIFFER, MOMI, INPAC). In some cases, policy-makers facilitated contacts or provided financial support for local adolescent SRH networks (CERCA).

## Discussion

The findings from the literature review and the interviews coincide in determining what the important theoretical elements are to ensure an optimal involvement of policy-makers: contacting them as early as possible in the project (including at proposal writing stage), ensuring regular communication, and making sure their participation is active by providing opportunities for feedback and input. The fact that activities to translate research into policies were included in all four FP7 projects reaffirms this finding. ICRH project coordinators were familiar with these requirements from previous experience. However, all projects met challenges that proved significant obstacles to the realization of the projects’ objectives.

The scarce literature available on policy-makers’ involvement in SRH projects warns that the sensitive nature of the topic might represent an obstacle in mobilizing this audience [3, 5–7]. This was illustrated by DIFFER, however only a few policy-makers expressed clear reluctance. Neither INPAC nor CERCA, although working on sensitive SRH topics (namely abortion, including in young unmarried women, and sexual and reproductive health in teenagers) experienced real difficulties. Policy-makers seemed to recognize the problem of teenage pregnancy (CERCA) and were willing to work in the frame of national regulations on post-abortion services (INPAC). This shows that policy-makers could be mobilized on sensitive SRH questions by finding a common language adapted to the local context (CERCA; [12, 19]). However, it may require from researchers to adopt their terminology, which raises questions on how far an agreement is actually reached between research and policy-makers (CERCA; [12]).

Interviews highlighted the presence of local partner organizations in the local and national SRH policy landscape, which is echoed in existing literature claiming that the credibility of scientific organizations and networks was crucial in motivating policy-makers to translate research findings into policy change [16]. Academic partners (CERCA) or partners with a significant experience in SRH (INPAC, MOMI, CERCA) were perceived by the coordinating teams as having better access to relevant and interested policy-makers. Moreover, the extent to which research processes and findings, beyond the immediate interventions, were acknowledged was determinant in how far policy-makers were willing to be involved (CERCA, DIFFER). It is interesting to note that when occurrences of this finding were found in the literature – which was rare - it was in studies focusing on SRH [5, 13]. This seems to support the idea that evidence may be perceived very differently by researchers and policy-makers [16].

Ensuring regular and quality contacts required significant investments in time and efforts from both project coordinating teams and local consortium partners, a challenge that is widely recognized in the literature [7, 12]. It sometimes conflicted with the necessity to implement and monitor the interventions to reach the projects’ objectives. This issue was heightened at sites facing structural challenges (staff turnover and workload, distance, lack of incentives), which seems to indicate that translational activities are particularly difficult to achieve in resource-constrained environments and thus in LMICs. This is a finding that reinforces the need, expressed in the literature, of knowing more on translation processes in LMICs [12, 16, 18].

Three of the four projects described here are still being implemented and therefore we are not able to discuss meaningfully their results in terms of policy impact, particularly in the long-term. CERCA has obtained results in terms of policy-makers’ involvement and policy change at various levels – these results are described in this article published by members of the consortium [19]. Follow-up is needed to see how the evidence is being used in the various contexts in which the projects were/are being implemented.

## Conclusions

We aimed at bringing researchers’ own experiences with translating research into policies together with current knowledge on these processes, a perspective that has rarely been explored in the existing literature [12]. Although the current knowledge on policy-makers’ involvement in SRH research outside North American and European contexts, particularly in LMICs, remains limited, it appears that the main objectives of translational activities remain the same in different settings. However, there are major challenges stemming from local contexts which should be addressed by SRH projects in order to develop efficient strategies. Context-specific strategies are key, even though they might sometimes not be enough to address important structural issues in policy-making processes. Therefore, future projects should specifically look into the possible local challenges and address these according to their resources as early as possible in the project life cycle.

## Additional file

Additional file 1: Interview Guide. (DOCX 15 kb)

## Abbreviations

CERCA: Community-Embedded Reproductive Health Care for Adolescents in Latin America
COREQ: Consolidated criteria for reporting qualitative studies
DIFFER: Diagonal interventions to fast forward enhanced reproductive health care
EC: European Commission
FP7: Seventh Framework Programme
ICRH: International Centre for Reproductive Health
INPAC: Integrating Post-Abortion Family Planning Services into Existing Abortion Services in Hospital Settings in China
LMICs: Low- and middle-income countries
MOMI: Missed Opportunities in Maternal and Infant Health
PABs: Policy Advisory Boards
SRH: Sexual and reproductive health
