# Supplementary material for: Practical lessons for bringing policy-makers on board in sexual and reproductive health research
Source: BMC Health Serv Res. 2016 Nov 11;16:649. doi: 10.1186/s12913-016-1889-1 (PMC5106764; doi:10.1186/s12913-016-1889-1)
Supplement: Additional file 1: — Interview Guide. (DOCX 15 kb) [file 12913_2016_1889_MOESM1_ESM.docx]

**Questionnaire – FP7 projects**

Building the project activities

1/ Why did you deem it important to involve stakeholders from the start of the project?

2/ Before starting this project, what was for you particularly important to consider when dealing with policy-makers?

3/ How did you select the activities to include in the project regarding translation of research results into policy *(previous experiences, similar projects at ICRH, literature review,etc…)*?

4/ Do you feel your background influences your perception of translation processes?

During the project

*Relational*

5/ In this project, what is your and your partners’ experience with involving stakeholders in research about SRH?

6/ Have you or field partners already felt that policy-makers were especially difficult to approach and/or convince because the research is about SRH *(in general or specific SRH topics in the project)*?

*Project management*

7/ Do you (and partners) find the time to develop stakeholder involvement-related activities or are they often pushed behind day-to-day project activities? Please explain.

8/ What are the major obstacles regarding stakeholders involvement encountered in the field?

9/ What is your current strategy *(you and partners)* to overcome those obstacles?

10/ Is there anything *(ex: better knowledge of political systems)* you feel might help you and/or your partners improve your approach? If yes, please explain. If no, please explain.

Lessons learned

11/ What do you find most difficult in involving stakeholders and translating research into policy?

12/ What do you find most rewarding in involving stakeholders and translating research into policy?

13/ After your experience with this project, what would you conclude is the most important aspect to consider for success in translation processes?
